# The Role of *Porphyromonas gingivalis* Lipopolysaccharide (PG-LPS) in Advanced Periodontitis—A Scoping Review

**DOI:** 10.3390/antibiotics15090886

**Published:** 2026-09-09

**Authors:** Tim Klomp, Andreas Braun, Georg Conrads

**Affiliations:** 1 Department of Operative Dentistry, Periodontology and Preventive Dentistry, Rheinisch-Westfälische Technische Hochschule University Hospital, 52074 Aachen, Germany; 2 Division of Oral Microbiology and Immunology, Department of Operative Dentistry, Periodontology and Preventive Dentistry, Rheinisch-Westfälische Technische Hochschule University Hospital, 52074 Aachen, Germany

**Keywords:** *Porphyromonas gingivalis*, advanced periodontitis, lipopolysaccharides (LPS), Toll-like receptor (TLR), outer membrane vesicles (OMV)

## Abstract

**Background**: *Porphyromonas gingivalis* is a Gram-negative, anaerobic keystone pathogen strongly implicated in the pathogenesis of periodontitis. Its pathogenicity is mediated through virulence factors, such as lipopolysaccharides (PG-LPS), which have been reported to display conflicting results regarding structural composition and immunogenicity. **Objectives:** This scoping review investigates how PG-LPS modulates host immunity and contributes to advanced periodontitis, with emphasis on its structural composition, divergent immunogenic properties, and the possible regulatory factors underlying these observations. Furthermore, PG-LPS interactions with periodontal tissues and associations with outer membrane vesicles (OMV) are examined. **Materials and Methods**: A structured literature search was conducted in PubMed following the PRISMA-ScR guidelines and PCC frameworks. **Results**: Structural heterogeneity, especially within the lipid A moiety of PG-LPS, accounts for previously inconsistent findings regarding its immunogenicity. Minor alterations in lipid A composition through enzymatic dephosphorylation or deacylation profoundly affect Toll-like receptor (TLR) engagement, antimicrobial resistance, and cytokine induction, and contribute to the biogenesis of OMVs. Environmental factors such as hemin availability and temperature influence enzymatic activity, resulting in lipid A isoforms that act as TLR4 agonists, antagonists, or remain immunologically inert. This structural plasticity mediates dynamic immune modulation that fosters polymicrobial dysbiosis and perpetuates non-resolving inflammation, leading to progressive tissue destruction, while concurrently contributing to the systemic inflammatory burden. **Conclusions**: Lipid A heterogeneity represents a central adaptive strategy potentially linking environmental cues and the bioenergetic status of *P. gingivalis* to immunomodulative properties. Verification of in vivo lipid A structural shifts in clinical isolates could inform novel therapeutic approaches targeting *P. gingivalis*-mediated inflammation.

## 1. Introduction

Periodontitis represents one of the most prevalent chronic inflammatory diseases and remains a major cause of tooth loss in adults worldwide. Most recent epidemiological data from Germany (DMS VI) [1] demonstrate improvements in oral health, including reduced prevalence of deep periodontal pockets and tooth loss, alongside an overall increase in tooth retention among older adults. Nevertheless, probing depths ≥4 mm remain unchanged, indicating that severe periodontitis persists as a major challenge, particularly in the aging population where demand for periodontal care is expected to rise [2]. This phenomenon, described as “morbidity compression” [3], reflects a shift in disease burden toward later life stages. On a global scale, however, the prevalence of periodontitis has shown little improvement since 1990, highlighting the urgent need for more effective preventive strategies and public health interventions to mitigate its impact [4,5].

Periodontitis is sustained by a complex interplay between dysbiotic subgingival biofilms and a dysregulated and non-resolving host inflammatory response [6,7,8]. In its advanced stages, periodontitis leads to the progressive destruction of gingival connective tissue, periodontal ligament, and alveolar bone, which significantly impairs oral function and quality of life [8,9]. Beyond the oral cavity, studies have linked periodontitis with several systemic diseases [10,11], including diabetes mellitus, cardiovascular disease, rheumatoid arthritis, and neurodegenerative conditions [12], underscoring its relevance as a public health concern [13].

Among the diverse microbiota of the periodontal pocket, *Porphyromonas gingivalis*, a rod-shaped to pleomorphic, non-motile, Gram-negative, obligate anaerobe, opportunistic pathogenic bacterium, has been established as a keystone pathogen [14], exerting a disproportionate influence on the microbial community and host response despite its relatively low abundance [15]. Furthermore, *P. gingivalis* is frequently detected in deep periodontal pockets of patients with severe periodontitis and has been consistently associated with increased levels of pro-inflammatory mediators such as interleukin−1β (IL−1β) and matrix metalloproteinase−8 (MMP−8), both of which correlate with alveolar bone resorption and disease severity [16,17,18]. *P. gingivalis* utilizes a broad array of virulence factors, such as fimbriae, gingipains, and outer membrane vesicles (OMVs) [19]. OMVs are spherical extracellular structures released by Gram-negative bacteria that transport diverse bioactive molecular cargo, including lipopolysaccharides and other virulence factors, thereby facilitating interbacterial communication and host–pathogen interactions [20]. By circumventing host defenses, they contribute to bacterial pathogenicity and promote polymicrobial persistence within the periodontal niche [21,22]. Of particular interest are the lipopolysaccharides (LPS), which play a pivotal role in host–pathogen interactions and constitute key determinants of immune modulation. Structurally, these macromolecules consist of a lipid A moiety, a core oligosaccharide, and an O-antigen polysaccharide [23]. Their biological activity is primarily attributed to the lipid A moiety, which anchors the molecule within the outer membrane of Gram-negative bacteria [24]. Pathogenic effects are mediated once LPS is released via OMV formation, cell division, or bacterial lysis [8,23]. Accordingly, free LPS function as potent endotoxins that elicit strong host immune responses [8]; at low concentrations LPS withhold the capacity to “awaken” and “train” a dormant immune system [25], while excessive levels of LPS can result in severe systemic effects, including high fever, hypotension, vascular coagulation, and, ultimately, lethal shock [8] Overall, LPS molecules are recognized as microbe-associated molecular patterns (MAMPs) that engage host pattern recognition receptors (PRRs), primarily Toll-like receptors (TLRs) [8].

Aberrant TLR signaling has been implicated in various pathological conditions, including chronic inflammatory diseases, thereby identifying them as a nexus between microbial attack and dysregulated, non-resolving inflammation within the host [24,26]. In *Escherichia coli*, the lipid A moiety is typically hexa-acylated and bis-phosphorylated, conferring strong agonistic activity on TLR4. Upon binding to the TLR4–MD−2–CD14 complex, this canonical endotoxin activates NF-κB signaling and inflammasome pathways, leading to the production of pro-inflammatory cytokines such as tumor necrosis factor α (TNF−α), interleukin−1β (IL−1β), and interleukin−6 (IL−6), as well as chemokines, including interleukin−8 (IL−8) and monocyte chemoattractant protein−1 (MCP−1). This robust TLR4 agonism of *E. coli* LPS has long served as the paradigm for endotoxin-mediated immune activation [27,28].

By contrast, the PG-LPS diverges significantly from this model. While it retains the tripartite structure typical of Gram-negative bacteria, early investigations into PG-LPS characterized it as an atypical endotoxin with immunological properties distinct from those of enterobacterial LPS, leading to initial controversy regarding its principal host-recognition pathway and inflammatory potential. Subsequent structural analyses revealed substantial heterogeneity in the lipid A moiety, including differences in acylation and phosphorylation that can confer TLR4 agonistic, antagonistic, or weakly immunogenic activity. Consequently, conflicting and sometimes contradictory findings exist in the literature regarding the structural composition and immunogenic properties of PG-LPS. Diverging reports suggest activation of either TLR4 or TLR2, with outcomes ranging from pro-inflammatory signaling to immune suppression [28]. This structural plasticity has established PG-LPS as an important determinant of *P. gingivalis* immune evasion and persistence and has provided a potential mechanistic link between bacterial adaptation, dysbiosis, and the chronic inflammatory response characteristic of periodontitis.

The molecular mechanisms by which PG-LPS influences the host immune response and contributes to the pathogenesis of advanced periodontitis, as well as structural heterogeneity among PG- LPS and factors underlying these structural alterations of bacterial LPS, will be further investigated in this review. Another objective is to describe the interaction between PG-LPS and periodontal tissue and resulting changes in pro-inflammatory mediators. Furthermore, possible correlations between PG- LPS and the formation of OMVs and their role in advanced periodontitis will be addressed. In this context, elucidating the role of PG-LPS in advanced periodontitis contributes to a deeper understanding of host–pathogen interactions that could inform promising avenues for developing targeted therapeutic strategies.

## 2. Results

**Figure 1:**
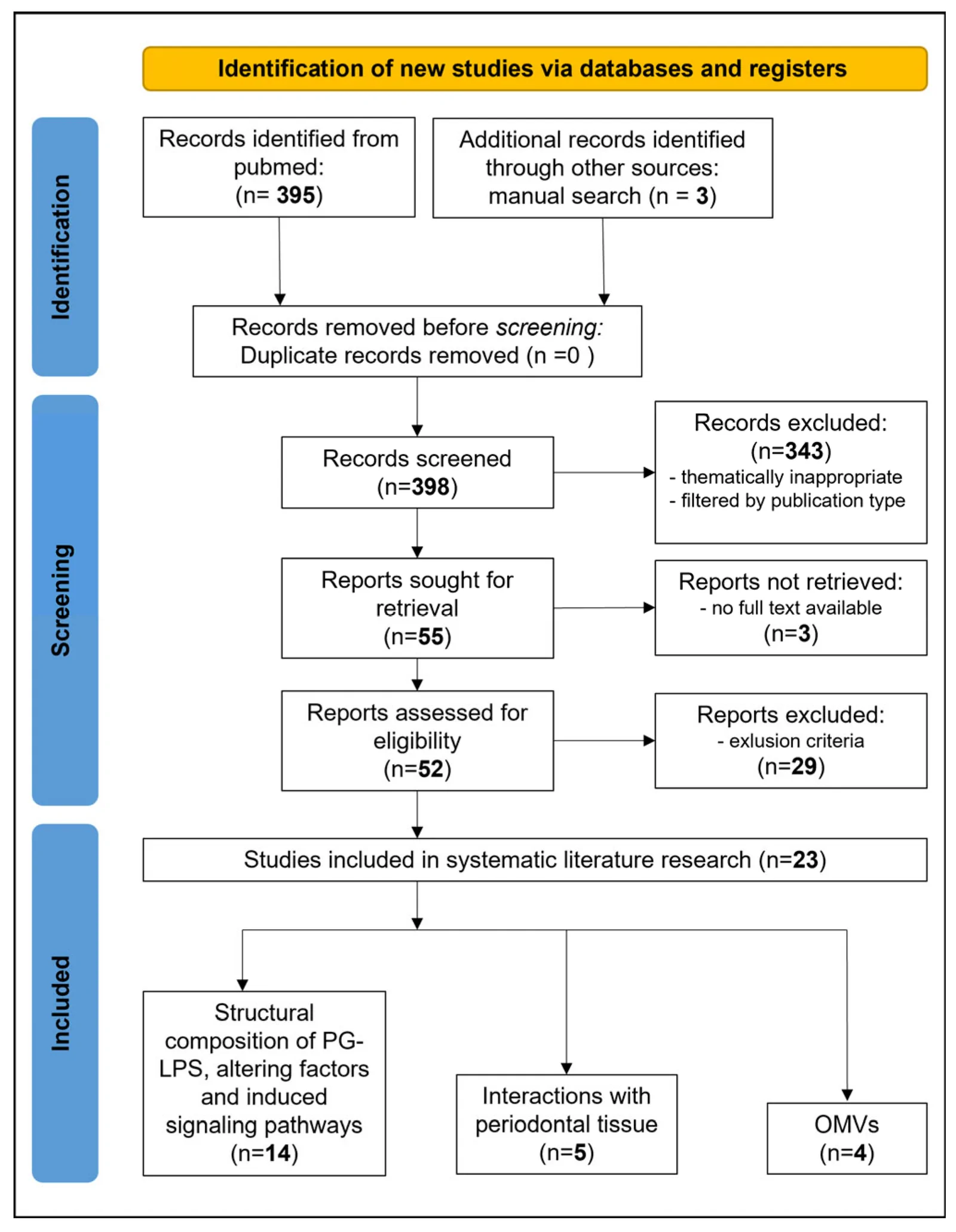
PRISMA flow chart modified according to Page et. al. 2021 [29].

The results of the literature search and study selection process are summarized in the PRISMA flow chart in Figure 1.

A total of 395 records were identified from PubMed and an additional three reports from a manual search during the first screening; 343 records were excluded after screening, and another three reports were excluded because no full text could be retrieved. The remaining 52 articles were assessed for eligibility. After the removal of 29 reports due to exclusion criteria, a total of 23 studies were categorized and included in this review. Among these studies, 10 reviews and 13 experimental studies were allocated to three different thematic sub-categories.

The first category includes studies concerning the structural composition of PG-LPS, TLR interactions, and factors that are associated with an alteration of PG-LPS structure. A total of 14 articles, including eight reviews [21,28,30,31,32,33,34,35] and six experimental studies [36,37,38,39,40,41], were included in this category. The next sub-category includes one review [42] and four experimental studies [43,44,45,46] that address the interactions between PG-LPS and periodontal tissue. Lastly, one review [47] and three different experimental studies [48,49,50] revolving around the OMVs of *P. gingivalis* were included in the final sub-aspect. A summary of included sources of evidence can be found in the Supplementary Materials, Tables S1 and S2.

### 2.1. Structural Composition of PG-LPS, TLR-Interactions, and Factors That Are Associated with an Alteration of PG-LPS Structure

#### 2.1.1. Structural Composition of PG-LPS

Structural heterogeneity of PG-LPS is primarily determined by modifications within the lipid A moiety, which consists of a β(1–6)-linked D-glucosamine disaccharide backbone [51], variably phosphorylated and acylated by long-chain branched fatty acids. Earlier reports identified tetra- and penta-acylated lipid A structures as predominant forms [52], which have been confirmed by several work groups since then [21,28,30,33,34,35,36,37,38,39,41,45,53]. One notable exception describes PG-LPS as containing a predominantly single tri-acylated variant (*m*/*z* 1195) [51]. The reasons for this apparent discrepancy are not clear but may involve culture conditions, strain variation, or isolation procedures. Although discrepancies exist concerning precise phosphorylation and acylation patterns, the consensus supports that these structural variants collectively underpin the immunological diversity and virulence potential of *P. gingivalis*.

**Figure 2:**
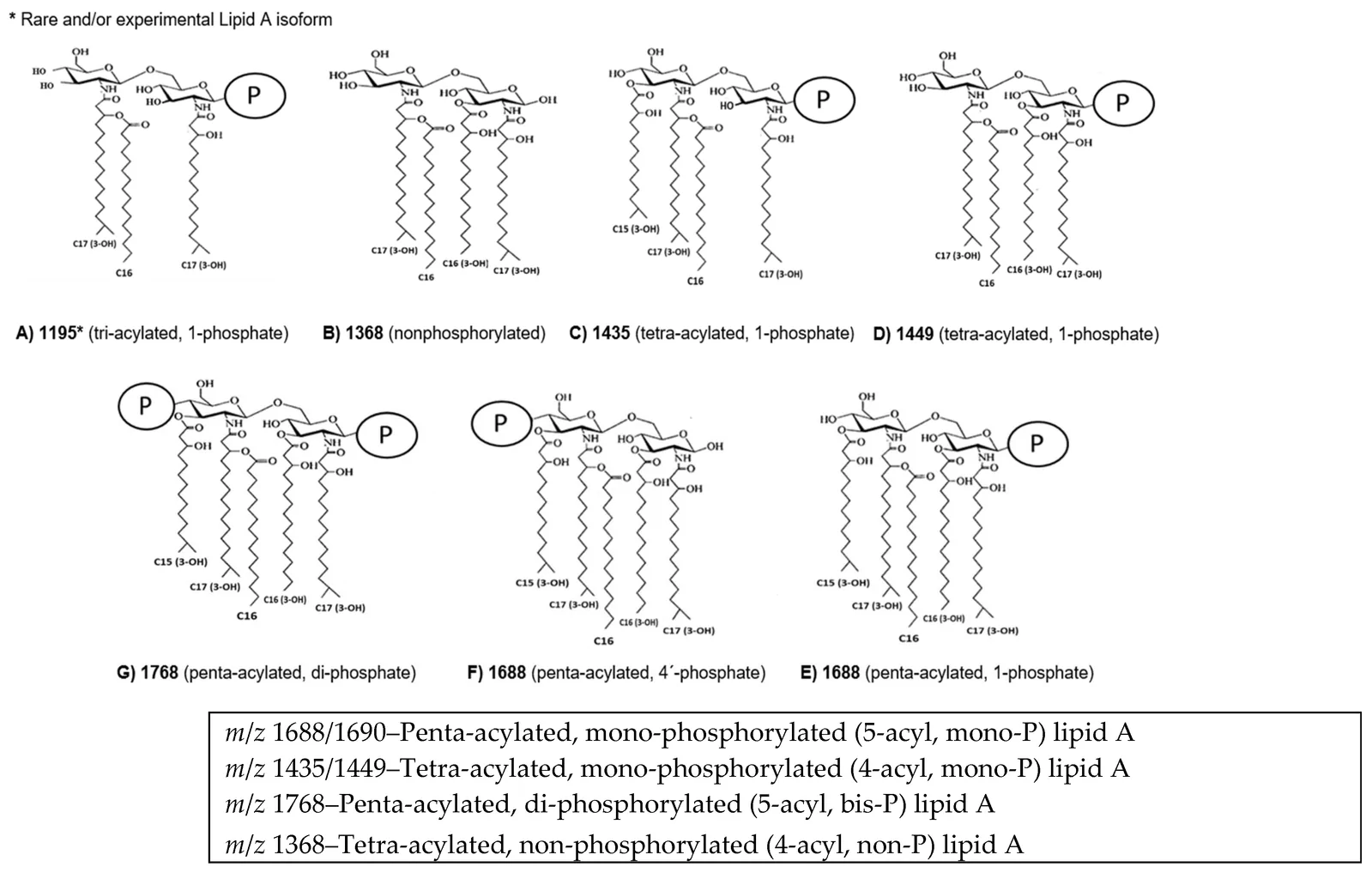
Structural variants of *P. gingivalis* lipid A displaying different acylation patterns and phosphorylation status. Numbers indicate the molecular mass weight of each lipid A variant. The structural variants were initially elucidated by [52]. Figure modified according to [28,36,39].

Compared to the uniform hexa-acylated *E. coli* lipid A (*m*/*z* 1797) [28], *P. gingivalis* lipid A displays marked heterogeneity, forming distinct clusters at *m*/*z* 1368, 1435/1449, 1688/1690, and 1768 [28,34]. These correspond to tetra- and penta-acylated species with variable phosphorylation states, including tetra-acylated non- or mono-phosphorylated and penta-acylated mono- or bis-phosphorylated forms [37,39,53]. Tetra-acylated lipid A has been labeled LPS_1435/1449_ based on its molecular weight of 1435 and 1449 Da. Accordingly, LPS_1688/1690_ reflects the molecular weight of 1688/1690 Da in designated lipid A structures. Non-phosphorylated tetra-acylated lipid A with a molecular weight of 1368 Da has been reported by five different groups [21,28,37,41,53]. Bis-phosphorylated penta-acylated lipid A with a molecular weight of 1768 Da was reported by six independent work groups [28,36,37,39,41,53]. An overview of identified lipid A isoforms is listed below and illustrated in Figure 2. 

According to Marcano et al. [33], two major PG-LPS forms have been described, O-LPS and A-LPS, which are distinguished by their polysaccharide composition and provide another level of structural heterogeneity within PG-LPS. O-LPS contains a tetrasaccharide O-antigen typical of Gram-negative bacteria and exhibits strain-specific antigenic variability within *P. gingivalis* [32,42]. In contrast, A-LPS is composed of anionic polysaccharide repeats covalently linked to lipid A [42]. Importantly, structural heterogeneity has been reported for A-LPS [33]. Further reports suggest that *P. gingivalis* uniquely co-expresses A-LPS and glycosylated Arg-gingipains, which act synergistically as immune effectors [54]. Mutants lacking A-LPS due to deletions in *porR* (PG1138) or *wbpB* (PG2119) exhibit reduced gingipain activity and increased susceptibility to complement-mediated killing, confirming the role of A-LPS in serum resistance [55] and suggesting a connection between investigated gene loci and A-LPS biosynthesis [55]. In the non-pigmented strain HG66, a *wbpB* mutation correlates with A-LPS deficiency, further supporting this association [55].

#### 2.1.2. Immunomodulative Properties and Altering Factors of LPS Variants

The immunomodulatory properties of *P. gingivalis* lipid A are determined by acylation and phosphorylation patterns that define its interaction with TLR4. The tetra-acylated, non-phosphorylated lipid A (*m*/*z* 1368) is TLR4-inert, promoting immune evasion, whereas the tetra-acylated, mono-phosphorylated form (*m*/*z* 1435/1449) acts as a TLR4 antagonist, mediating immune suppression. In contrast, penta-acylated mono- and bis-phosphorylated species (*m*/*z* 1688 and 1768) serve as TLR4 agonists [28,36].

Environmental conditions, particularly hemin concentration, profoundly modulate lipid A biosynthesis [32,36,56]. High hemin availability—typical of inflamed periodontal sites—promotes conversion of lipid A toward an immunosuppressive/TLR4-antagonistic phenotype. Conversely, low hemin conditions favor the expression of non-phosphorylated, tetra-acylated lipid A species [28,36,53]. Temperature also influences lipid A composition: growth at 41 °C induces a shift toward the more immunostimulatory penta-acylated form, while temperatures of 37 °C favor tetra-acylated lipid A isoforms [37].

#### 2.1.3. Genetic Determinants of Lipid A Structural Heterogeneity

**Figure 3:**
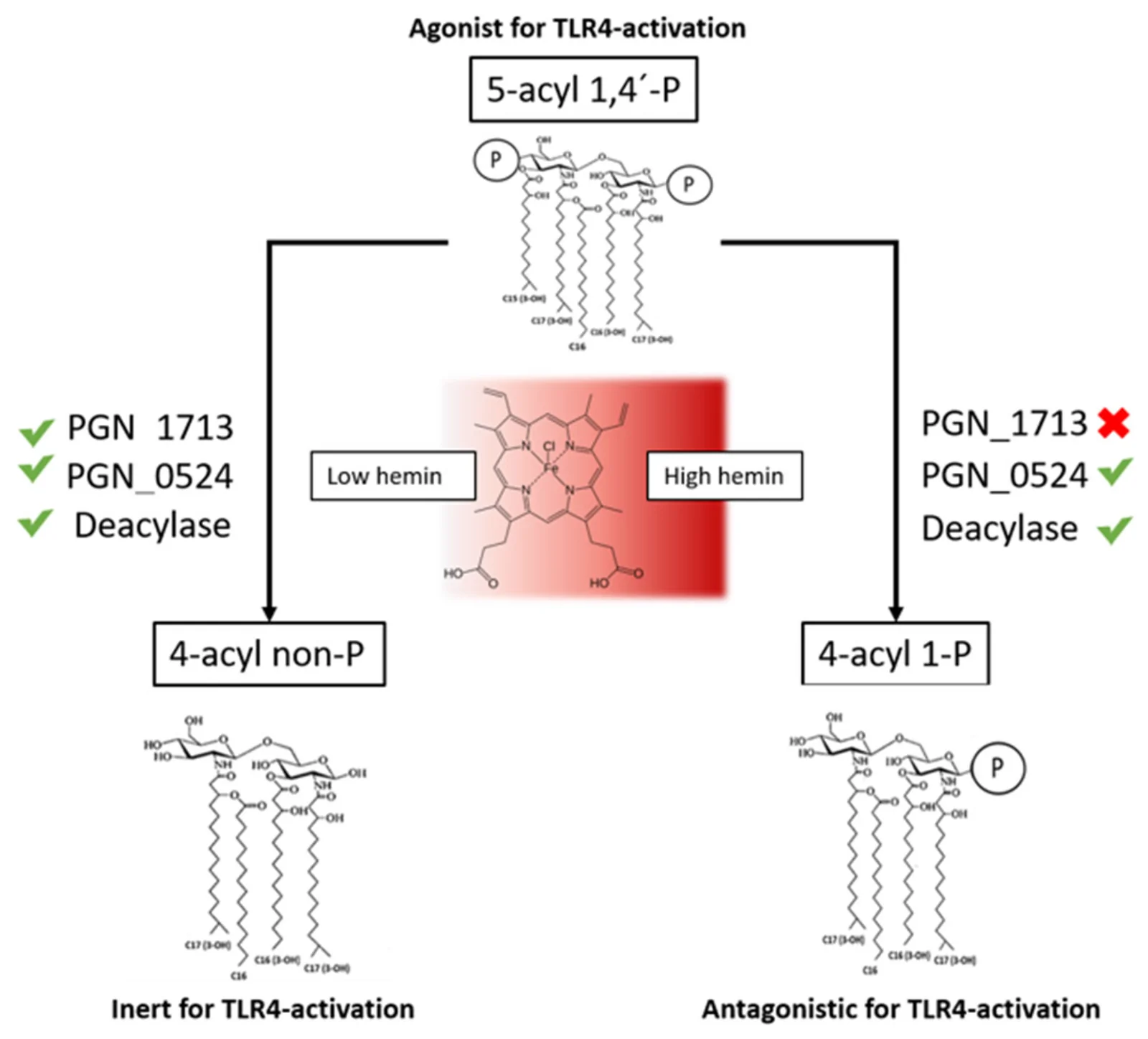
A model of differential 1- and 4’-phosphatase activities, encoded by PGN_1713 and PGN_0524, respectively, under varying hemin concentrations. Under high hemin conditions, the activity of the lipid A 1-phosphatase (PGN_1713) is proposed to be inhibited. Figure modified with permission according to [53].

Lipid A structural diversity in *P. gingivalis* results from post-synthetic remodeling of a penta-acylated, bis-phosphorylated precursor (*m*/*z* 1768) through the action of specific lipid A phosphatases and deacylases [53]. These enzymes sequentially remove phosphate and acyl groups, generating mono- and non-phosphorylated, as well as tetra-acylated variants [28,53]. Figure 3 offers a schematic overview of enzymatic activities resulting in different lipid A isoforms under different hemin levels.

Two key phosphatases have been identified: PGN_1713 (C1-phosphatase) and PGN_0524 (C4’-phosphatase). Inactivation of PGN_1713 leads to the accumulation of tetra-acylated, mono-phosphorylated lipid A (*m*/*z* 1449), mirroring the antagonist profile of wild-type *P. gingivalis* under high hemin conditions. Conversely, PGN_0524 deletion results in penta-acylated, mono-phosphorylated lipid A (*m*/*z* 1688), which acts as a moderate TLR4 agonist [53]. Double mutants lacking both enzymes accumulate bis-phosphorylated penta-acylated lipid A—the most potent TLR4 agonistic isoform identified in *P. gingivalis* [53].

Notably, the ΔPGN_0524 mutant produces very little tetra-acylated lipid A, indicating that C4′-phosphate removal is a prerequisite for the loss of an acyl chain from the penta-acylated precursor. This observation aligns with similar findings in other bacterial species [28,53]. Sequential phosphate removal precedes deacylation and governs the bacterium’s switch between immunostimulatory and immuno-evasive phenotypes.

The activity of these phosphatases is environmentally regulated [36,37,39]. High hemin concentrations inhibit 1-phosphatase activity, promoting the formation of antagonistic lipid A species, while elevated temperature (≥41 °C) or loss of 4′-phosphatase function favors agonistic variants [21,37]. Importantly, while penta-acylated lipid A species are detectable by MALDI-TOF MS and thin-layer chromatography, their functional role is likely overshadowed by tetra-acylated lipid A species, which exhibit either an inert phenotype under low hemin conditions or an antagonistic effect under high hemin conditions in wild-type *P. gingivalis* [53].

A recent hypothesis proposed by Ghods et al. [39] suggests that intracellular signaling via second messengers, particularly cyclic di-adenosine monophosphate (c-di-AMP), may regulate lipid A–modifying phosphatases at either the transcriptional or post-translational level. Within *P. gingivalis*, several key components of the c-di-AMP signaling network were identified, including the c-di-AMP synthase gene (*dacPg*, PGN_0523), the c-di-AMP phosphodiesterase gene (*pdePg*, PGN_0521), and the predicted transcriptional regulator *cdaR* (PGN_1486). These gene loci regulate intracellular c-di-AMP signaling, linking pyruvate availability to lipid A modification. Perturbation of this signaling system disrupts the synthesis of penta-acylated mono-phosphorylated species and alters glycosylation, notably reducing N-acetylgalactosamine (GalNAc) incorporation [39]. Given that GalNAc is suggested to modify LPS antigenicity, its reduction may decrease host immune recognition and modulate TLR4 signaling [39].

#### 2.1.4. TLR-Activation Bias

*P. gingivalis* lipid A primarily engages the TLR4 complex, initiating pro-inflammatory signaling via NF-κB and cytokine production [28,57]. However, the assertion that PG-LPS can also engage TLR2 has been a controversy in the field. TLR2 interaction was attributed to unique lipid A features of PG-LPS such as long acyl chains, reduced acylation pattern and phosphorylation status, or contamination by lipoproteins and other bacterial lipids [32,58,59]. Subsequent studies with chemically synthesized *P. gingivalis* lipid A analogues confirmed exclusive TLR4 activation, demonstrating that tri-, tetra-, and penta-acylated species activate macrophages and fibroblasts via TLR4, but not TLR2 [34,57,60]. TLR2 activation, according to some in vitro and in vivo models, might primarily be driven by *P. gingivalis* surface-associated lipoproteins rather than LPS itself [42,61]. Conversely, in a ligature-induced murine model, TLR2—but not TLR4—mediates pathological bone resorption, highlighting tissue-specific and receptor-dependent inflammatory responses [62]. Co-culture studies suggest that both TLR2 and TLR4 may contribute to osteoclast activation in vitro, whereas in vivo bone resorption is largely TLR2-dependent [43,63].

*P. gingivalis* LPS exhibits competitive antagonism at the TLR4-MD−2 complex, attenuating responses to potent agonists from other bacteria, such as *Aggregatibacter actinomycetemcomitans*. This antagonistic effect results from binding of antagonistic lipid A species to MD−2, thereby preventing agonist lipid A engagement and modulating host inflammatory responses [28,31,53].

#### 2.1.5. Resistance to Antimicrobial Peptides

Lipid A structural modifications critically determine *P. gingivalis* susceptibility to cationic antimicrobial peptides (CAMPs), including polymyxin B. Polymyxin B is a cationic cyclic peptide that disrupts the bacterial outer membrane via electrostatic interactions with negatively charged lipopolysaccharides. Resistance is conferred by lipid A dephosphorylation or substitution of phosphate groups with positively charged moieties, reducing electrostatic binding [28,64]. In *P. gingivalis*, the prevalent non-phosphorylated, tetra- and penta-acylated lipid A species confer high resistance, allowing growth at polymyxin B concentrations up to 200 μg/mL. In contrast, a ΔPGN_0524_ mutant, which is unable to generate non-phosphorylated lipid A, is highly sensitive, with growth inhibited at 2 μg/mL [41,64]. These findings indicate that the coordinated activity of lipid A phosphatases and deacylases provides a substantial survival advantage against CAMPs, including physiologically relevant antimicrobial peptides such as β-defensins and cathelicidins [21,28,37,53].

### 2.2. Interactions Between PG-LPS and Periodontal Tissue

The interaction of PG-LPS with host periodontal tissues reveals a complex and cell type-specific modulation of immune and inflammatory responses [35]. Signaling pathways have already been shown to be related to the structural composition of *P. gingivalis* lipid A isomers and the targeted cells. In gingival fibroblasts and epithelial cells, the tetra-acylated LPS_1435/1449_ and penta-acylated LPS_1690_ variants interact with both TLR2 and TLR4 in a cell type- and context-dependent manner [30,42]. As already outlined, LPS_1690_ acts as a potent TLR4 agonist, strongly activating NF-κB signaling and inducing transcription of pro-inflammatory mediators, including IL−6, IL−8, GM-CSF, CXCL10, and CCL2 [65]. In contrast, LPS_1435/1449_ displays weak agonistic or even antagonistic activity toward TLR4, resulting in suppressed NF-κB activation and reduced cytokine expression [42,65]. Additionally, LPS_1690_ upregulates synthesis of molecules related to inflammation, oxidative stress, and cytoskeletal reorganization, such as iNOS, galectin, cathepsins, and mitochondrial antioxidant enzymes, whereas LPS_1435/1449_ preferentially induces anti-inflammatory mediators, including Annexin A2 and A6 [45]. Moreover, fibroblasts derived from chronically inflamed periodontal tissues exhibit a reduced cytokine response to PG-LPS [44]. Epithelial cells exposed to these LPS forms exhibit altered expression of β-defensins and adhesion molecules, ultimately facilitating epithelial barrier dysfunction and matrix degradation [32].

LPS_1435/1449_ was found to evade noncanonical inflammasome activation, promoting intracellular bacterial persistence, while LPS_1690_ triggered inflammasome activation and IL−1β secretion, thereby reducing bacterial survival [35,41] in macrophages. PG-LPS induces the production of pro-inflammatory cytokines, including IL−1α, IL−1β, IL−6, IL−8, IL−18, and TNF-α, in monocytes under in vitro conditions [32,33]. Furthermore, *P. gingivalis* interferes with host autophagy to support intracellular persistence. LPS_1690_ induces extensive LC3-positive autophagosome formation, whereas LPS_1435_/_1449_ inhibit this process, reducing autophagic degradation [66].

The study by Akkaoui et al. [43] demonstrated that aging significantly reduces osteoclast precursor (OCP) responsiveness to PG-LPS due to altered TLR expression and signaling. OCPs from aged mice exhibited decreased TLR2 and TLR4 levels and a diminished capacity for tartrate-resistant acid phosphatase-positive (TRAP^+^) osteoclast formation following RANKL and PG-LPS stimulation. In contrast, OCPs from young mice responded robustly via TLR4-mediated pathways, while TLR2 inhibition had no effect. Elevated SASP-associated cytokines (TNF-α, IL−1β, IL−6) and increased RANKL expression in young mice further enhanced osteoclastogenic activity [43].

In endothelial cells, LPS_1690_ induces E-selectin expression, thereby promoting endothelial activation and leukocyte adhesion [42]. Again, the tetra-acylated LPS_1435/1449_ functions as a TLR4 antagonist, effectively suppressing *E. coli* LPS-induced E-selectin (ELAM-1) expression through direct interference with the TLR4 receptor ectodomain [53]. Comparative analyses using human and chimeric TLR4 models have demonstrated that these lipid A variants interact with TLR4 signaling complexes in structurally distinct manners [56]. Exposure of endothelial cells to PG-LPS induces a dose-dependent modulation of ICAM-1, VCAM-1, and E-selectin [67].

In human oral keratinocytes, recombinant human lipopolysaccharide-binding protein (rhLBP) alone was reported to induce IL−6 and IL−8 expression primarily through TLR2-mediated activation of NF-κB, JNK/p38 MAPK, and IRF signaling pathways [38]. However, when LBP interacts with PG-LPS variants, differential regulatory effects are observed. Both LPS_1435/1449_ and LPS_1690_ suppress LBP-induced IL−6 expression, with the tetra-acylated LPS_1435/1449_ exhibiting a stronger inhibitory effect than the penta-acylated LPS_1690_ [38]. Conversely, LPS_1690_ upregulates LBP expression in keratinocytes via combined TLR2 and TLR4 activation, thereby amplifying the potential for subsequent receptor-mediated responses [42].

Studies on animal cementoblasts (OCCM-30) have demonstrated that PG-LPS induces MMP expression [33]. Furthermore, PG-LPS_1690_ significantly upregulated NF-κB transcription via TLR2, whereas *E. coli* LPS exerted its effects through TLR4 [30,68].

Notably, Slocum et al. [41] reported site-specific vascular inflammation for distinct lipid A moieties. As reported above, LPS_1435/1449_ facilitates immune evasion by suppressing TLR4-mediated pro-inflammatory signaling and inhibiting noncanonical inflammasome activation, thereby promoting bacterial persistence within macrophages [35,41]. This strategy reduces cytokine production, prevents pyroptosis, and preserves an intracellular niche, while parallel activation of TLR2 through lipoproteins, fimbriae, and phosphorylated dihydroceramides sustains chronic low-grade inflammation [41]. In experimental models, strains producing LPS_1435/1449_ exacerbated vascular inflammation and accelerated atherosclerosis in ApoE(-/-) mice, whereas strains restricted to agonistic lipid A triggered inflammasome activation, host cell lysis, and diminished systemic inflammation due to reduced bacterial survival [41]. These observations suggest that lipid A remodeling differentially governs local and systemic disease: periodontal bone loss progresses independently of lipid A status, while systemic inflammatory outcomes are tightly linked to antagonistic TLR4 signaling [41,42].

### 2.3. Outer Membrane Vesicles in the Context of PG-LPS

*P. gingivalis* releases LPS both through bacterial lysis and via outer membrane vesicles [30,69]. These OMVs were reported to penetrate gingival tissues, disrupt TLR4-dependent antimicrobial responses, and suppress epithelial β-defensin expression, potentially protecting other bacteria within the same mixed-species biofilm [21,31]. In addition to LPS, OMVs carry gingipains, fimbriae, and other virulence factors that enhance immune evasion and tissue destruction [21]. Comparative analyses of OMVs from red-complex pathogens demonstrated that *P. gingivalis* is the most prolific vesicle producer, eliciting broad activation of multiple PRRs, whereas *Tannerella forsythia* and *Treponema denticola* induced weaker or minimal responses [48]. Structural differences in OMV composition among these periodontopathogens correspond to distinct host PRR interactions [48].

The immune responses triggered by PG-OMVs differ from those induced by whole bacteria or free LPS: PG-OMVs have been shown to elicit strong TLR2- and TLR4-mediated responses in vitro [48]. In epithelial cells and fibroblasts, vesicles induce IL−6 and IL−8 production via activation of multiple signaling pathways, including NF-κB, MAPK, and STING [50]. Furthermore, PG-OMVs have been shown to suppress fibroblast and endothelial cell proliferation in a dose-dependent manner and inhibit angiogenesis in vitro, disrupting tissue repair [47]. In osteoclast precursor cells, PG-OMVs enhance differentiation and expression of osteoclastogenic cytokines, predominantly via TLR2 activation [49]. Moreover, OMVs disrupt vascular function by upregulating the expression of endothelial adhesion molecules, such as E-selectin, thereby promoting leukocyte adhesion and vascular inflammation [47].

The presence of distinct LPS structures within OMVs appears to be essential for vesicle biogenesis. Specifically, dephosphorylation of lipid A in the anionic LPS (A-LPS) region, mediated by the outer membrane protein PG0027 [70], has been shown to destabilize the bacterial membrane and promote vesicle blebbing [42]. Notably, the structural curvature induced by desphosphorylated LPS further facilitates OMV formation [47], while CTD (C-terminal domain) family proteins display structural compatibility with A-LPS, resulting in their incorporation into the vesicle surface [47,69,70].

## 3. Discussion

### 3.1. Structural Heterogeneity Among PG-LPS

The findings of this review underline the pivotal role of PG-LPS as a major virulence determinant in advanced periodontitis. Lipid A heterogeneity is not an experimental artifact but a biologically relevant feature that enables *P. gingivalis* to finely modulate host immune responses. Structural plasticity among *P. gingivalis* lipid A, especially the ability to shift towards a TLR4-antagonistic isoform, underlies its ability to modulate host immune activation and resist antimicrobial clearance by host defenses [21,28,30,31,32,33,34,35,36,37,38,39,53,56,57].

Overall, two levels of lipid A heterogeneity were identified. The first involves a relaxed substrate specificity of acyltransferases LpxA and LpxD, which incorporate fatty acids of variable chain length into the nascent lipid A, potentially accounting for observed “clusters” of lipid A structural variants in the MALDI-TOF spectrum [36,53].

Additionally, discrepancies in reported lipid A structures across studies were largely attributed to methodological factors, including differences in extraction techniques, purification steps, and mass spectrometry parameters. Phenol-water extraction, for instance, was shown to selectively enrich specific lipid A variants, whereas MgCl_2_–EtOH-based methods introduced additional biases. Synthetic or commercial PG-LPS preparations, such as SMB00610 (Sigma-Aldrich Lipopolysaccharide from *Porphyromonas gingivalis*, Merck, Darmstadt, Germany), are widely employed in experimental studies; however, they represent only a TLR4-agonistic isoform. Therefore, many in vitro assays do not adequately reflect the full spectrum of structural and functional heterogeneity within PG-LPS. Co-purified lipoproteins and lipid contaminants further complicated mass spectrometric profiles, emphasizing the necessity for standardized extraction and analytical procedures to ensure comparability between studies. The recently introduced FLATn/LPSpure approach enables precise in situ lipid A profiling [36,39]. By refining analytical techniques such as FLATn-MS, researchers can achieve a more precise characterization of lipid A structures in clinical samples, unraveling their immunological implications in periodontal disease [28].

The second level of heterogeneity concerns differences in acyl chain number and phosphate positioning, influenced by environmental factors [34,36,37,38,39,53]. Hemin availability was shown to regulate virulence-associated traits, including gingipain expression and OMV formation [71]. Temperature elevations enhanced the synthesis of TLR4-agonistic lipid A forms, linking environmental adaptation to inflammatory potential [30,36,37,53].

Beyond lipid A variability, the association between synthesis pathways of A-LPS and Arg-gingipains (a toxic protease) suggests a coordinated regulation of endotoxin and exotoxin activity [54]. Theoretically, this observation, alongside reports suggesting the involvement of specific hemin-sensing proteins such as Kgp and HmuR [53] in the adaptive remodeling of lipid A in response to environmental hemin fluctuations, supports the plausibility of a comparable regulatory mechanism governing endotoxin and exotoxin activity. Especially since both effectors seem to play a role in OMVs [47]. Shared biosynthetic pathways may enable *P. gingivalis* to balance immune activation and tissue destruction through a dynamic interplay between LPS-mediated inflammation and gingipain-mediated proteolysis [28,42,72].

*P. gingivalis* employs structural remodeling of lipid A via enzymatic modifications, mediated by phosphatases (PGN_1713/PG1773 and PGN_0524/PG1587) and putative deacylases [37,53,64,73]. Notably, second messengers such as cyclic-di-AMP may regulate these phosphatases at the transcriptional or post-translational level. As Ghods et al. (2024) [39] suggested, c-di-AMP signaling could integrate environmental inputs, such as hemin availability and exogenous pyruvate, to regulate lipid A composition. In *P. gingivalis*, an elevated bioenergetic status leads to naturally occurring antagonism via ablation of phosphatase activity. In this context, the term bioenergetics refers to the study of the flow and transformation of energy from substrate to ATP in living organisms. The ability to fine-tune its lipid A structure in response to environmental cues represents a sophisticated mechanism that reflects a nexus between the bioenergetic status of the cell, resistance against antimicrobial peptides, and immunomodulative properties [28,36,37,42,64].

In essence, the dual effect—immune evasion through TLR4 antagonism and decreased susceptibility to host- or drug-derived cationic peptides—represents a potent survival strategy that is sought to facilitate immune evasion and promote dysbiosis in a polymicrobial biofilm, ultimately contributing to the chronic inflammatory characteristics observed in advanced periodontitis. These mechanisms underline the critical role of lipid A heterogeneity in periodontal pathogenesis and highlight the need for standardized analytical approaches and targeted therapeutic strategies aimed at disrupting immunomodulation in advanced periodontitis.

### 3.2. Interactions on a Tissue Level

PG-LPS was reported to manipulate TLR4–mediated NF-κB signaling in human gingival fibroblasts with isoform-dependent effects ranging from the amplification of local inflammation and connective tissue destruction to antagonistic immunomodulative effects and impairment of tissue repair [33,56,65], illustrating a context-dependent balance [45,65]. In a comparable manner, PG-LPS has divergent effects on the integrity of endothelial cells [42,67], which may facilitate bacterial dissemination and contribute to vascular inflammation, possibly linking periodontal infection to systemic pathologies [33]. Collectively, PG-LPS acts as a multifaceted modulator of cell type-specific inflammatory responses, ultimately favoring connective tissue breakdown and impaired repair processes. PG-LPS promotes osteoclastogenesis, and age-dependent modulation of TLR signaling might affect bone turnover in periodontitis [43]. Further studies revealed that PG-LPS-induced bone resorption can occur via the TLR4/TNF-α/TNFR-2 axis independently of the canonical RANKL/RANK/OPG pathway. These findings suggest that interfering with TLR4-mediated cytokine cascades may represent a viable therapeutic approach for mitigating inflammatory bone loss in periodontitis and other osteolytic conditions [74]. Notably, observations by Slocum et. al. [41] suggest that lipid A remodeling differentially governs local and systemic disease: periodontal bone loss progresses independently of lipid A status, while systemic inflammatory outcomes are tightly linked to antagonistic TLR4 signaling [41,42].

### 3.3. Possible Correlations Between PG-LPS and the Formation of OMVs

OMVs represent critical mediators in the pathogenesis of periodontitis and serve as vehicles for local and systemic dissemination of virulence factors. The biogenesis of these “micro-bullets” [69] is regulated by lipid A dephosphorylation mediated by PG0027, which destabilizes the outer membrane and facilitates vesicle formation, predominantly in A-LPS-enriched regions [70]. Selective incorporation of anionic LPS and CTD-family proteins enhances OMV pathogenicity, enabling efficient delivery of virulence factors into host tissues [47]. On a cellular level, OMVs promote vascular inflammation [75], impair periodontal wound healing by degrading integrin-associated signaling molecules [47], and contribute to alveolar bone resorption [49] and immune evasion by inhibiting TLR4-dependent β-defensin expression and macrophage bactericidal activity, offering bystander protection that facilitates bacterial persistence [21,31]. Beyond local effects, *P. gingivalis* OMVs participate in biofilm development and nutrient acquisition, particularly heme sequestration, which benefits both *P. gingivalis* and the polymicrobial community [47]. Increasing evidence links OMV-mediated dissemination of endotoxins to systemic inflammatory conditions, including atherosclerosis, rheumatoid arthritis, diabetes, and neurodegenerative diseases [69,76]. Chronic exposure to LPS-containing OMVs induces low-grade systemic inflammation, thereby providing a plausible link between periodontitis and systemic comorbidities.

Despite growing evidence, inconsistencies in experimental approaches and cell models have limited mechanistic understanding of OMV biogenesis and cargo selection. Further research is required to clarify the regulatory interplay between LPS heterogeneity, OMV formation, and immune modulation. Unraveling these mechanisms could provide the foundation for novel therapeutic strategies targeting OMV production or function, potentially mitigating both periodontal and systemic inflammatory burden.

Taken together, several complementary perspectives have been proposed regarding the role of PG-LPS in periodontitis. While some studies support a predominantly pro-inflammatory role through TLR-dependent activation of innate immune pathways, others suggest that structurally distinct lipid A species exert TLR4-antagonistic or immunologically inert effects, thereby facilitating immune evasion and bacterial persistence. The available evidence therefore supports a context-dependent model in which the immunological activity of PG-LPS is determined by its lipid A structure and influenced by bacterial strain, environmental conditions, and experimental methodology, rather than by a uniform pro- or anti-inflammatory activity. Furthermore, the evidence underscores a complex network through which PG-LPS orchestrates immune modulation across multiple periodontal cell types. Structural heterogeneity of lipid A determines receptor engagement, cytokine output, and antimicrobial sensitivity, whereas contextual cues such as hemin availability, temperature, and overall bioenergetic status dictate which LPS variant predominates. The cumulative effect is a shift from acute to chronic, non-resolving inflammation, sustaining microbial persistence and progressive tissue destruction. By selectively activating or inhibiting TLR-dependent pathways, PG-LPS modulates the host immune response in a manner that promotes microbial dysbiosis and contributes to the progression of periodontal disease.

### 3.4. Clinical Implications

The observed structural and immunomodulatory heterogeneity of PG-LPS may have translational implications for both therapeutic and diagnostic approaches in periodontitis. Modulation of PG-LPS–TLR signaling could represent a potential adjunctive therapeutic strategy, provided that protective antimicrobial responses are preserved. Furthermore, FLATn-MS-based profiling of agonistic, antagonistic, and immunologically inert lipid A isoforms in saliva or gingival crevicular fluid may provide a molecular readout/biomarker of *P. gingivalis* adaptation and its influence on the local inflammatory environment, with potential utility for assessing disease activity, progression, or treatment response. Longitudinal clinical studies are required to determine whether specific lipid A signatures can complement established clinical and microbiological parameters for periodontal risk stratification and therapeutic monitoring.

### 3.5. Study Limitations

Some limitations should be considered when interpreting the findings of this scoping review. Although the review was designed according to PRISMA-ScR guidelines, only 23 publications were identified over approximately two decades, potentially reflecting a restrictive search strategy and limiting the comprehensiveness of the available evidence. In addition, some aspects of the PRISMA-ScR guidelines, such as the availability of a pre-registered protocol, additional databases as sources of information, a critical appraisal of some of the extracted data, and the inclusion of independent investigators in the data charting process, are not fulfilled, thus representing further limitations. Furthermore, substantial methodological heterogeneity among the reviewed studies complicates direct comparison and interpretation. Differences in *P. gingivalis* strains, culture conditions, LPS extraction and purification procedures, and experimental models may influence lipid A structure and its reported immunological activity. In particular, challenges associated with LPS isolation and the potential for contamination with lipoproteins or other bacterial components may contribute to divergent findings regarding TLR2- and TLR4-mediated responses. Moreover, many studies employed highly controlled in vitro conditions that do not fully reflect the complex polymicrobial and inflammatory environment of the periodontal pocket. Variations in LPS concentrations, cell types, experimental endpoints, and analytical methods further limit comparability. Accordingly, the available evidence should be interpreted cautiously, and the contribution of specific PG-LPS structures to periodontal pathogenesis cannot yet be established conclusively. Standardized approaches to bacterial strain characterization, LPS purification, structural analysis, and functional immune assays are warranted to clarify the role of PG-LPS structural heterogeneity in immune modulation and periodontitis.

### 3.6. Research Gaps and Future Perspectives

Several areas require further investigation to clarify the role of PG-LPS in advanced periodontitis. First, standardized protocols for LPS extraction and purification, as well as lipid A structural analysis, are required to improve comparability between studies and minimize potential confounding by lipoproteins or other bacterial components. Within this context, the proposed FLATn-MS-method seems to offer promising diagnostic potential.

Secondly, the impact of environmental factors, such as hemin availability or temperature, on lipid A remodeling, and the resulting immunological activity, should be investigated under physiologically relevant conditions. Exploring therapeutic interventions targeting lipid A remodeling enzymes, or regulatory circuits such as c-di-AMP signaling, represents promising avenues to disrupt immune evasion and restore periodontal homeostasis while maintaining antibiotic stewardship. Thirdly, well-characterized individual lipid A species should be examined in standardized cellular and in vivo models to distinguish TLR2- and TLR4-dependent effects and to determine their relative contributions to inflammatory signaling, immune evasion, inflammasome activation, and autophagy. Furthermore, the role of PG-LPS-containing outer membrane vesicles in the dissemination and biological activity of distinct LPS structures warrants further investigation. Overall, longitudinal and clinically relevant studies are needed to determine how PG-LPS structural heterogeneity changes during periodontal disease progression and treatment and how these changes relate to dysbiosis, periodontal tissue destruction, and potential systemic effects. Addressing these research gaps would contribute to resolving the current inconsistencies in the literature and establish a more comprehensive understanding of PG-LPS as a modulator of host–microbe interactions in periodontitis.

## 4. Materials and Methods

A structured scoping literature review was conducted to investigate the role of PG-LPS in advanced periodontitis. The search strategy was designed largely in accordance with the PRISMA-ScR guidelines [77]. To formulate the research question and define the scope of the review, the PCC model was applied [29,78]. The literature search was performed in PubMed, supplemented by a manual search to identify additional relevant studies. The literature search was conducted on 5 February 2025. The eligibility criteria were defined as follows: Inclusion criteria comprised studies published in English or German with full-text availability, directly addressing the role of PG-LPS in advanced periodontitis. Eligible publication types included meta-analyses, systematic reviews, narrative reviews, randomized controlled trials, clinical studies, and experimental studies, thereby ensuring both clinical and mechanistic perspectives. Exclusion criteria were defined as studies not referring to PG-LPS, investigations focused on implant-related pathologies, diseases other than periodontitis, or therapeutic interventions not directly related to PG-LPS. The timeframe was restricted to the last 20 years to reflect trends and advances in the field. The complete search query, the PRISMA-ScR checklist and two additional tables containing the study characteristics of all included sources of evidence can be seen in the Supplementary Materials.

This structured approach enabled the evaluation of all sub-aspects relevant to the objective, including PG-LPS heterogeneity, its immunological properties, its contribution to periodontal tissue interactions, and outer membrane vesicle formation. A narrative synthesis of the extracted data was produced. In line with the methodology recommendations for scoping reviews, a formal assessment of the risk of bias in the studies was not performed [77].

## 5. Conclusions

In advanced periodontitis, PG-LPS can play a dual role—while actively modulating immune responses to evade host defense, it withholds the potential to drive chronic inflammation, tissue destruction, and bone resorption [24,32]. Its unique structural adaptability is sought to enable it to persist, disrupt immune homeostasis, and possibly contribute to systemic disease progression [79,80]. Considering these multifaceted effects, PG-LPS is a critical determinant in the progression of periodontitis, shifting the host–microbe balance toward a chronic inflammatory state that supports *P. gingivalis* survival while driving periodontal destruction [21,31]. It has been shown that *P. gingivalis* exhibits the remarkable ability to synthesize diverse lipid A structures in response to specific environmental conditions [28,30,36,37,39,53]. These lipid A variants can function as TLR4 agonists, antagonists, or remain biologically inert. The presence of TLR4-antagonistic lipid A has the potential to disrupt the finely tuned balance of innate immune mediators typically produced in response to commensal bacteria, thereby altering host–microbiome interactions [21,28,31]. Such immune modulation may have far-reaching consequences, not only for the pathogenesis of periodontitis but also for broader microbial community dynamics within the host. Despite its significance, the mechanisms by which bacterial modulation of immune mediator production contributes to destructive inflammation remain unclear. Further research in this area is essential to deepen our understanding of periodontitis and its potential links to other chronic inflammatory diseases.

## Data Availability

No new data were created or analyzed in this study. Data sharing is not applicable to this article.

## Supplementary Materials

The following supporting information can be downloaded at: https://www.mdpi.com/article/10.3390/antibiotics15090886/s1, Figure S1: Search query using MeSH terms and Boolean operators for the scoping literature research in PubMed; Table S1: Overview of included reviews after literature search; Table S2: Overview of included experimental studies after literature search. Table S3: PRISMA-ScR Checklist.

## Abbreviations

The following abbreviations are used in this manuscript:

PG-LPS	*Porphyromonas gingivalis* lipopolysaccharide
LPS	Lipopolysaccharide
OMV	Outer membrane vesicles
TLR	Toll-like receptor
PRR	Pattern recognition receptor
MAMP	Microbe-associated molecular pattern
S	Structure of PG-LPS
TI	Tissue interaction
AMR	Antimicrobial resistance
OCP	Osteoclast precursor cell
hGFs	Human gingival fibroblasts
WT	Wild-type
CI	Clinical isolates
mAB	Monoclonal antibodies
HOK	Human oral keratinocytes
CP	Chronic periodontitis

## References

[1] Eickholz P., Holtfreter B., Kuhr K., Dannewitz B., Jordan A.R., Kocher T. (2025). Prevalence of the periodontal status in Germany: Results of the 6th German Oral Health Study (DMS • 6). Quintessence Int..

[2] Schützhold S., Kocher T., Biffar R., Hoffmann T., Schmidt C.O., Micheelis W., Jordan R., Holtfreter B. (2015). Changes in prevalence of periodontitis in two German population-based studies. J. Clin. Periodontol..

[3] Jordan A.R., Bodechtel C., Hertrampf K., Hoffmann T., Kocher T., Nitschke I., Schiffner U., Stark H., Zimmer S., Micheelis W. (2014). The Fifth German Oral Health Study (Fünfte Deutsche Mundgesundheitsstudie, DMS V)—Rationale, design, and methods. BMC Oral Health.

[4] Nascimento G.G., Alves-Costa S., Romandini M. (2024). Burden of severe periodontitis and edentulism in 2021, with projections up to 2050: The Global Burden of Disease 2021 study. J. Periodontal Res..

[5] Wu L., Zhang S.-Q., Zhao L., Ren Z.-H., Hu C.-Y. (2022). Global, regional, and national burden of periodontitis from 1990 to 2019: Results from the Global Burden of Disease study 2019. J. Periodontol..

[6] Bartold P.M., van Dyke T.E. (2019). An appraisal of the role of specific bacteria in the initial pathogenesis of periodontitis. J. Clin. Periodontol..

[7] Meyle J., Chapple I. (2015). Molecular aspects of the pathogenesis of periodontitis. Periodontol. 2000.

[8] Wolf H.F., Rateitschak E.M., Rateitschak K.H. (2012). Parodontologie.

[9] Agnese C.C.D., Schöffer C., Kantorski K.Z., Zanatta F.B., Susin C., Antoniazzi R.P. (2025). Periodontitis and Oral Health-Related Quality of Life: A Systematic Review and Meta-Analysis. J. Clin. Periodontol..

[10] Deschner J. (2018). Interaktionen zwischen Parodontitis und systemischen Erkrankungen. Freie Zahnarzt.

[11] Mei F., Xie M., Huang X., Long Y., Lu X., Wang X., Chen L. (2020). *Porphyromonas gingivalis* and Its Systemic Impact: Current Status. Pathogens.

[12] Bregaint S., Boyer E., Fong S.B., Meuric V., Bonnaure-Mallet M., Jolivet-Gougeon A. (2022). *Porphyromonas gingivalis* outside the oral cavity. Odontology.

[13] Genco R.J., Sanz M. (2020). Clinical and public health implications of periodontal and systemic diseases: An overview. Periodontol. 2000.

[14] Hajishengallis G., Darveau R.P., Curtis M.A. (2012). The keystone-pathogen hypothesis. Nat. Rev. Microbiol..

[15] Socransky S.S., Haffajee A.D., Cugini M.A., Smith C., Kent R.L. (1998). Microbial complexes in subgingival plaque. J. Clin. Periodontol..

[16] Müller B.E. (2020). Untersuchung zur Korrelation Zwischen der Klinischen Manifestation der Marginalen Parodontitis und der Expression von Matrixmetalloproteinase 8 und Surfactant Protein D im Serum. Ph.D. Thesis.

[17] Griffen A.L., Lyons S.R., Becker M.R., Moeschberger M.L., Leys E.J. (1999). *Porphyromonas gingivalis* strain variability and periodontitis. J. Clin. Microbiol..

[18] Chigasaki O., Aoyama N., Sasaki Y., Takeuchi Y., Mizutani K., Ikeda Y., Gokyu M., Umeda M., Izumi Y., Iwata T. (2021). *Porphyromonas gingivalis*, the most influential pathogen in red-complex bacteria: A cross-sectional study on the relationship between bacterial count and clinical periodontal status in Japan. J. Periodontol..

[19] Mysak J., Podzimek S., Sommerova P., Lyuya-Mi Y., Bartova J., Janatova T., Prochazkova J., Duskova J. (2014). *Porphyromonas gingivalis*: Major periodontopathic pathogen overview. J. Immunol. Res..

[20] Magaña G., Harvey C., Taggart C.C., Rodgers A.M. (2023). Bacterial Outer Membrane Vesicles: Role in Pathogenesis and Host-Cell Interactions. Antibiotics.

[21] Hajishengallis G., Diaz P.I. (2020). *Porphyromonas gingivalis*: Immune subversion activities and role in periodontal dysbiosis. Curr. Oral Health Rep..

[22] Hajishengallis G., Lamont R.J. (2021). Polymicrobial communities in periodontal disease: Their quasi-organismal nature and dialogue with the host. Periodontol. 2000.

[23] Schromm A.B., Brandenburg K., Loppnow H., Moran A.P., Koch M.H., Rietschel E.T., Seydel U. (2000). Biological activities of lipopolysaccharides are determined by the shape of their lipid A portion. Eur. J. Biochem..

[24] Bainbridge B.W., Darveau R.P. (2001). *Porphyromonas gingivalis* lipopolysaccharide: An unusual pattern recognition receptor ligand for the innate host defense system. Acta Odontol. Scand..

[25] Spear G.T., Marshall P., Teodorescu M. (1986). Increase in proliferation and cytotoxic cell development in human mixed lymphocyte cultures in the presence of very low concentrations of LPS: Role of IL-1 and prostaglandin E2. Clin. Immunol. Immunopathol..

[26] Czerkies M., Kwiatkowska K. (2014). Toll-Like Receptors and their Contribution to Innate Immunity: Focus on TLR4 Activation by Lipopolysaccharide. Adv. Cell Biol..

[27] Pulendran B., Kumar P., Cutler C.W., Mohamadzadeh M., van Dyke T., Banchereau J. (2001). Lipopolysaccharides from distinct pathogens induce different classes of immune responses in vivo. J. Immunol..

[28] Jain S., Darveau R.P. (2010). Contribution of *Porphyromonas gingivalis* lipopolysaccharide to periodontitis. Periodontol. 2000.

[29] Page M.J., McKenzie J.E., Bossuyt P.M., Boutron I., Hoffmann T.C., Mulrow C.D., Shamseer L., Tetzlaff J.M., Akl E.A., Brennan S.E. (2021). The PRISMA 2020 statement: An updated guideline for reporting systematic reviews. BMJ.

[30] Jia L., Han N., Du J., Guo L., Luo Z., Liu Y. (2019). Pathogenesis of Important Virulence Factors of *Porphyromonas gingivalis* via Toll-Like Receptors. Front. Cell. Infect. Microbiol..

[31] Hajishengallis G., Krauss J.L., Liang S., McIntosh M.L., Lambris J.D. (2012). Pathogenic microbes and community service through manipulation of innate immunity. Adv. Exp. Med. Biol..

[32] Bostanci N., Belibasakis G.N. (2012). *Porphyromonas gingivalis*: An invasive and evasive opportunistic oral pathogen. FEMS Microbiol. Lett..

[33] Marcano R., Rojo M.Á., Cordoba-Diaz D., Garrosa M. (2021). Pathological and Therapeutic Approach to Endotoxin-Secreting Bacteria Involved in Periodontal Disease. Toxins.

[34] Ogawa T., Yagi T. (2010). Bioactive mechanism of *Porphyromonas gingivalis* lipid A. Periodontol. 2000.

[35] Xu W., Zhou W., Wang H., Liang S. (2020). Roles of *Porphyromonas gingivalis* and its virulence factors in periodontitis. Adv. Protein Chem. Struct. Biol..

[36] Al-Qutub M.N., Braham P.H., Karimi-Naser L.M., Liu X., Genco C.A., Darveau R.P. (2006). Hemin-dependent modulation of the lipid A structure of *Porphyromonas gingivalis* lipopolysaccharide. Infect. Immun..

[37] Curtis M.A., Percival R.S., Devine D., Darveau R.P., Coats S.R., Rangarajan M., Tarelli E., Marsh P.D. (2011). Temperature-dependent modulation of *Porphyromonas gingivalis* lipid A structure and interaction with the innate host defenses. Infect. Immun..

[38] Ding P.-H., Darveau R.P., Wang C.-Y., Jin L. (2017). 3LPS-binding protein and its interactions with *P. gingivalis* LPS modulate pro-inflammatory response and Toll-like receptor signaling in human oral keratinocytes. PLoS ONE.

[39] Ghods S., Muszyński A., Yang H., Seelan R.S., Mohammadi A., Hilson J.S., Keiser G., Nichols F.C., Azadi P., Ernst R.K. (2024). The multifaceted role of c-di-AMP signaling in the regulation of *Porphyromonas gingivalis* lipopolysaccharide structure and function. Front. Cell. Infect. Microbiol..

[40] Kim P.D., Xia-Juan X., Crump K.E., Abe T., Hajishengallis G., Sahingur S.E. (2015). Toll-Like Receptor 9-Mediated Inflammation Triggers Alveolar Bone Loss in Experimental Murine Periodontitis. Infect. Immun..

[41] Slocum C., Coats S.R., Hua N., Kramer C., Papadopoulos G., Weinberg E.O., Gudino C.V., Hamilton J.A., Darveau R.P., Genco C.A. (2014). Distinct lipid a moieties contribute to pathogen-induced site-specific vascular inflammation. PLoS Pathog..

[42] Olsen I., Singhrao S.K. (2018). Importance of heterogeneity in Porhyromonas gingivalis lipopolysaccharide lipid A in tissue specific inflammatory signalling. J. Oral Microbiol..

[43] Akkaoui J., Yamada C., Duarte C., Ho A., Vardar-Sengul S., Kawai T., Movila A. (2021). Contribution of *Porphyromonas gingivalis* lipopolysaccharide to experimental periodontitis in relation to aging. GeroScience.

[44] Fitzsimmons T.R., Ge S., Bartold P.M. (2018). Compromised inflammatory cytokine response to *P. gingivalis* LPS by fibroblasts from inflamed human gingiva. Clin. Oral Investig..

[45] Herath T.D.K., Darveau R.P., Seneviratne C.J., Wang C.-Y., Wang Y., Jin L. (2016). Heterogeneous *Porphyromonas gingivalis* LPS modulates immuno-inflammatory response, antioxidant defense and cytoskeletal dynamics in human gingival fibroblasts. Sci. Rep..

[46] Wara-aswapati N., Chayasadom A., Surarit R., Pitiphat W., Boch J.A., Nagasawa T., Ishikawa I., Izumi Y. (2013). Induction of toll-like receptor expression by *Porphyromonas gingivalis*. J. Periodontol..

[47] Gui M.J., Dashper S.G., Slakeski N., Chen Y.-Y., Reynolds E.C. (2016). Spheres of influence: *Porphyromonas gingivalis* outer membrane vesicles. Mol. Oral Microbiol..

[48] Cecil J.D., O’Brien-Simpson N.M., Lenzo J.C., Holden J.A., Chen Y.-Y., Singleton W., Gause K.T., Yan Y., Caruso F., Reynolds E.C. (2016). Differential Responses of Pattern Recognition Receptors to Outer Membrane Vesicles of Three Periodontal Pathogens. PLoS ONE.

[49] Kim H.Y., Song M.-K., Lim Y., Jang J.S., An S.-J., Kim H.-H., Choi B.-K. (2022). Effects of extracellular vesicles derived from oral bacteria on osteoclast differentiation and activation. Sci. Rep..

[50] Uemura Y., Hiroshima Y., Tada A., Murakami K., Yoshida K., Inagaki Y., Kuwahara T., Murakami A., Fujii H., Yumoto H. (2022). *Porphyromonas gingivalis* Outer Membrane Vesicles Stimulate Gingival Epithelial Cells to Induce Pro-Inflammatory Cytokines via the MAPK and STING Pathways. Biomedicines.

[51] Ogawa T. (1993). Chemical structure of lipid A from Porphyromonas (Bacteroides) gingivalis lipopolysaccharide. FEBS Lett..

[52] Kumada H., Haishima Y., Umemoto T., Tanamoto K. (1995). Structural study on the free lipid A isolated from lipopolysaccharide of *Porphyromonas gingivalis*. J. Bacteriol..

[53] Coats S.R., Jones J.W., Do C.T., Braham P.H., Bainbridge B.W., To T.T., Goodlett D.R., Ernst R.K., Darveau R.P. (2009). Human Toll-like receptor 4 responses to *P. gingivalis* are regulated by lipid A 1- and 4′-phosphatase activities. Cell. Microbiol..

[54] Paramonov N., Rangarajan M., Hashim A., Gallagher A., Aduse-Opoku J., Slaney J.M., Hounsell E., Curtis M.A. (2005). Structural analysis of a novel anionic polysaccharide from *Porphyromonas gingivalis* strain W50 related to Arg-gingipain glycans. Mol. Microbiol..

[55] Shoji M., Sato K., Yukitake H., Naito M., Nakayama K. (2014). Involvement of the Wbp pathway in the biosynthesis of *Porphyromonas gingivalis* lipopolysaccharide with anionic polysaccharide. Sci. Rep..

[56] Reife R.A., Coats S.R., Al-Qutub M., Dixon D.M., Braham P.A., Billharz R.J., Howald W.N., Darveau R.P. (2006). *Porphyromonas gingivalis* lipopolysaccharide lipid A heterogeneity: Differential activities of tetra- and penta-acylated lipid A structures on E-selectin expression and TLR4 recognition. Cell. Microbiol..

[57] Sawada N., Ogawa T., Asai Y., Makimura Y., Sugiyama A. (2007). Toll-like receptor 4-dependent recognition of structurally different forms of chemically synthesized lipid As of *Porphyromonas gingivalis*. Clin. Exp. Immunol..

[58] Jain S., Coats S.R., Chang A.M., Darveau R.P. (2013). A novel class of lipoprotein lipase-sensitive molecules mediates Toll-like receptor 2 activation by *Porphyromonas gingivalis*. Infect. Immun..

[59] Ogawa T., Asai Y., Makimura Y., Tamai R. (2007). Chemical structure and immunobiological activity of *Porphyromonas gingivalis* lipid A. Front. Biosci..

[60] Zhang Y., Gaekwad J., Wolfert M.A., Boons G.-J. (2008). Synthetic tetra-acylated derivatives of lipid A from *Porphyromonas gingivalis* are antagonists of human TLR4. Org. Biomol. Chem..

[61] Lasica A.M., Goulas T., Mizgalska D., Zhou X., de Diego I., Ksiazek M., Madej M., Guo Y., Guevara T., Nowak M. (2016). Structural and functional probing of PorZ, an essential bacterial surface component of the type-IX secretion system of human oral-microbiomic *Porphyromonas gingivalis*. Sci. Rep..

[62] Burns E., Bachrach G., Shapira L., Nussbaum G. (2006). Cutting Edge: TLR2 is required for the innate response to *Porphyromonas gingivalis*: Activation leads to bacterial persistence and TLR2 deficiency attenuates induced alveolar bone resorption. J. Immunol..

[63] Lin J., Bi L., Yu X., Kawai T., Taubman M.A., Shen B., Han X. (2014). *Porphyromonas gingivalis* exacerbates ligature-induced, RANKL-dependent alveolar bone resorption via differential regulation of Toll-like receptor 2 (TLR2) and TLR4. Infect. Immun..

[64] Zenobia C., Hasturk H., Nguyen D., van Dyke T.E., Kantarci A., Darveau R.P. (2014). *Porphyromonas gingivalis* lipid A phosphatase activity is critical for colonization and increasing the commensal load in the rabbit ligature model. Infect. Immun..

[65] Herath T.D.K., Wang Y., Seneviratne C.J., Darveau R.P., Wang C.-Y., Jin L. (2013). The expression and regulation of matrix metalloproteinase-3 is critically modulated by *Porphyromonas gingivalis* lipopolysaccharide with heterogeneous lipid A structures in human gingival fibroblasts. BMC Microbiol..

[66] Blasi I., Korostoff J., Dhingra A., Reyes-Reveles J., Shenker B.J., Shahabuddin N., Alexander D., Lally E.T., Bragin A., Boesze-Battaglia K. (2016). Variants of *Porphyromonas gingivalis* lipopolysaccharide alter lipidation of autophagic protein, microtubule-associated protein 1 light chain 3, LC3. Mol. Oral Microbiol..

[67] Andrukhov O., Steiner I., Liu S., Bantleon H.P., Moritz A., Rausch-Fan X. (2015). Different effects of *Porphyromonas gingivalis* lipopolysaccharide and TLR2 agonist Pam3CSK4 on the adhesion molecules expression in endothelial cells. Odontology.

[68] Nemoto E., Darveau R.P., Foster B.L., Nogueira-Filho G.R., Somerman M.J. (2006). Regulation of cementoblast function by *P. gingivalis* lipopolysaccharide via TLR2. J. Dent. Res..

[69] Singhrao S.K., Olsen I. (2018). Are *Porphyromonas gingivalis* Outer Membrane Vesicles Microbullets for Sporadic Alzheimer’s Disease Manifestation?. J. Alzheimer’s Dis. Rep..

[70] Rangarajan M., Aduse-Opoku J., Hashim A., McPhail G., Luklinska Z., Haurat M.F., Feldman M.F., Curtis M.A. (2017). LptO (PG0027) Is Required for Lipid A 1-Phosphatase Activity in *Porphyromonas gingivalis* W50. J. Bacteriol..

[71] Veith P.D., Luong C., Tan K.H., Dashper S.G., Reynolds E.C. (2018). Outer Membrane Vesicle Proteome of *Porphyromonas gingivalis* Is Differentially Modulated Relative to the Outer Membrane in Response to Heme Availability. J. Proteome Res..

[72] Lunar Silva I., Cascales E. (2021). Molecular Strategies Underlying *Porphyromonas gingivalis* Virulence. J. Mol. Biol..

[73] Jain S., Chang A.M., Singh M., McLean J.S., Coats S.R., Kramer R.W., Darveau R.P. (2019). Identification of PGN_1123 as the Gene Encoding Lipid A Deacylase, an Enzyme Required for Toll-Like Receptor 4 Evasion, in *Porphyromonas gingivalis*. J. Bacteriol..

[74] AlQranei M.S., Senbanjo L.T., Aljohani H., Hamza T., Chellaiah M.A. (2021). Lipopolysaccharide- TLR-4 Axis regulates Osteoclastogenesis independent of RANKL/RANK signaling. BMC Immunol..

[75] Zhang Z., Liu D., Liu S., Zhang S., Pan Y. (2020). The Role of *Porphyromonas gingivalis* Outer Membrane Vesicles in Periodontal Disease and Related Systemic Diseases. Front. Cell. Infect. Microbiol..

[76] Olsen I. (2021). *Porphyromonas gingivalis*-Induced Neuroinflammation in Alzheimer’s Disease. Front. Neurosci..

[77] Tricco A.C., Lillie E., Zarin W., O’Brien K.K., Colquhoun H., Levac D., Moher D., Peters M.D.J., Horsley T., Weeks L. (2018). PRISMA Extension for Scoping Reviews (PRISMA-ScR): Checklist and Explanation. Ann. Intern. Med..

[78] Blümle A., Lagrèze W.A., Motschall E. (2018). Systematische Literaturrecherche in PubMed: Eine Kurzanleitung. Anaesthesist.

[79] Hajishengallis G. (2015). Periodontitis: From microbial immune subversion to systemic inflammation. Nat. Rev. Immunol..

[80] Zenobia C., Darveau R.P. (2022). Does Oral Endotoxin Contribute to Systemic Inflammation?. Front. Oral Health.

