# Supplementary material for: The Role of Porphyromonas gingivalis Lipopolysaccharide (PG-LPS) in Advanced Periodontitis—A Scoping Review
Source: Antibiotics (Basel). 2026 Sep 9;15(9):886. doi: 10.3390/antibiotics15090886 (PMC13603459; doi:10.3390/antibiotics15090886)
Supplement: Supplementary file 1 [file antibiotics-15-00886-s001.zip › antibiotics-4496568-supplementary.pdf]

Supplementary Materials:

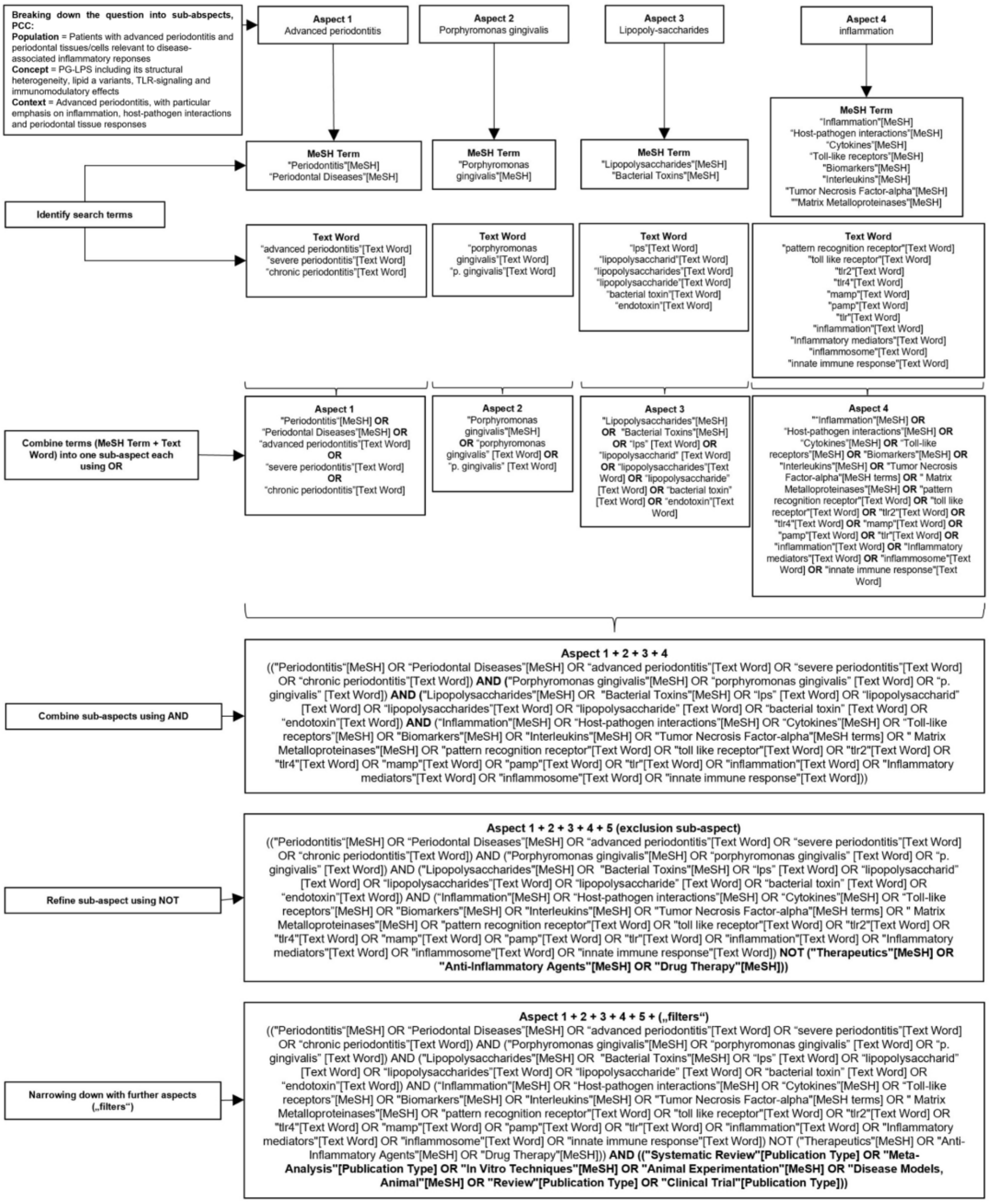

Figure S1 - Search query using MeSH terms and Boolean operators for the scoping literature research in PubMed

Table S1: Overview of included reviews after literature search

Author, Year	Type	Sub-aspect	Key Findings	Method	Strain	Cell culture/ Medium	LPS preparation	Animal model
**(Al-Qutub et al., 2006)**	In vitro	**S**	Environmental factors altering lipid A structure	LPS characterization via MALDI-TOF	ATCC 33277, W83, 381 and CI	TYHK and ETSB medium (+hemin)	Purification (Tri-reagent method and Phenol water procedure)	n.a.
**(Curtis et al., 2011)**	In vitro	**S**	Environmental factors altering lipid A structure, TLR signaling	LPS characterization via MALDI-TOF, TLR activation assay	W50, E.coli-LPS, HEK 293 cells	BHI (+hemin), DMEM medium	Purification (TRIzol-method)	n.a
**(Ding et al., 2017)**	In vitro	**S**	TLR signaling, Tissue interaction with LBP	LPS characterization via MALDI-TOF, ELISA, antiTLR2/4 (mAB)	ATCC 33277, HOK cells	n.a.	Purification (proteinase K digestion-method)	n.a.
**(Ghods et al., 2024)**	In vitro	**S**	Environmental factors and Genetic determinants altering lipid A structure, TLR signaling	LPS characterization via FLATn combined with MALDI-TOF, ELISA, TLR4 assay, SDS-PAGE and LPS staining + Western blot	381, 2 MS, HEK-Blue hTLR4 cells	TSA medium (+hemin), TSB liquid culture	Purification (proteinase k digestion-method)	n.a.
**(Kim et al., 2015)**	In vivo murine model and ex vitro	**S**	TLR signaling, Tissue interaction	(ex-vivo) ELISA, RT-PCR, Microscopic evaluation of BL	ATCC 33277	Splenocytes and bone marrow derived macrophages, BHI (+hemin)	n.a.	TLR9 knockout mice + WT
**(Slocum et al., 2014)**	In vitro experiment and in vivo murine model	**S**	TLR signaling, Tissue interaction, Genetic determinants of lipid A structural modifications	LPS characterization via MALDI-TOF, TLR activation assay, ELISA, in vivo MRA, Atherosclerotic plaque assessment and microcomputed tomography	381, 2 MS, HEK293 cells	Bone marrow derived macrophages, BHI (+hemin)	Purification	ApoE and C57BL/6 mice

Table S2: Overview of included experimental studies after literature search

Author, Year	Type	Sub-aspect	Key Findings	Method	Strain	Cell culture/ Medium	LPS preparation	Animal model
**(Akkaoui et al., 2021)**	In vivo murine model	**TI**	Tissue interaction, TLR signaling	ELISA for SASP markers, multicolor flow cytometry of OCPs	ATCC 33277, OCPs	Splenic monocytes (OCPs)	ultrapure LPS extraction	CSF1reGFP (KI) mice and WT C57BL/6
**(Fitzsimmons et al., 2018)**	In vitro	**TI**	Tissue interaction	Bead-based multiplex assay	ATCC 33277, hGFs	hGF cell culture (healthy and CP)	purified LPS extraction, *E.coli* LPS extraction (control)	n.a.
**(Herath et al., 2016)**	In vitro	**TI**	LPS heterogeneity, Tissue interaction	LPS characterization via MALDI-TOF, quantitative proteomics on hGFs	ATCC 33277, hGFs	hGF cell culture	Purification ($MgCl_2$-ethanol procedure), *E.coli* LPS extraction (control)	n.a.
**(Wara-aswapati et al., 2013)**	In vitro	**TI**	TLR signaling, Tissue interaction	Preparation of sonicated extract, TLR-gene-expression assessment via RT-PCR (+qPCR)	ATCC 33277, clinical samples (19 CP + control), hGFS	hGFs in cell culture	Purified LPS extraction	n.a.
**(Cecil et al., 2016)**	In vitro	**OMV**	OMV, TLR signaling, Tissue interaction	PRR assessment	*P.g., T.f.,* and *T.d.* (OMVs), HEK-Blue cells	n.a.	n.a.	n.a.
**(Kim et al., 2022)**	In vitro experiment and murine model	**OMV**	OMV, TLR signaling, Tissue interaction	OMV isolation, TLR assay, murine model for osteoclast differentiation	ATCC 33277, ATCC 43037, ATCC 35896, ATCC 9811, ATCC 23272, HEK Blue cells	n.a.	n.a.	yes
**(Uemura et al., 2022)**	In vitro experiment	**OMV**	OMV, Tissue interaction	OMV isolation, qRT-PCR, knockdown of STING mRNA in OBA-9 cells (siRNA)	ATCC 33277, OBA-9 cells	Human gingival epithelial cell line OBA-9	n.a.	n.a.

Table S3 : PRISMA-ScR Checklist

SECTION	ITEM	PRISMA-ScR CHECKLIST ITEM	REPORTED ON PAGE #
**TITLE**			
Title	1	Identify the report as a scoping review.	Page 1
**ABSTRACT**			
Structured summary	2	Provide a structured summary that includes (as applicable): background, objectives, eligibility criteria, sources of evidence, charting methods, results, and conclusions that relate to the review questions and objectives.	Page 1
**INTRODUCTION**			
Rationale	3	Describe the rationale for the review in the context of what is already known. Explain why the review questions/objectives lend themselves to a scoping review approach.	Page 2-3
Objectives	4	Provide an explicit statement of the questions and objectives being addressed with reference to their key elements (e.g., population or participants, concepts, and context) or other relevant key elements used to conceptualize the review questions and/or objectives.	Page 2-3; Supplementary Material Figure S1
**METHODS**			
Protocol and registration	5	Indicate whether a review protocol exists; state if and where it can be accessed (e.g., a Web address); and if available, provide registration information, including the registration number.	N/A
Eligibility criteria	6	Specify characteristics of the sources of evidence used as eligibility criteria (e.g., years considered, language, and publication status), and provide a rationale.	Page 14
Information sources*	7	Describe all information sources in the search (e.g., databases with dates of coverage and contact with authors to identify additional sources), as well as the date the most recent search was executed.	Page 14
Search	8	Present the full electronic search strategy for at least 1 database, including any limits used, such that it could be repeated.	Page 14, Supplementary Material FigureS1
Selection of sources of evidence†	9	State the process for selecting sources of evidence (i.e., screening and eligibility) included in the scoping review.	Page 14
Data charting process‡	10	Describe the methods of charting data from the included sources of evidence (e.g., calibrated forms or forms that have been tested by the team before their use, and whether data charting was done independently or in duplicate) and any processes for obtaining and confirming data from investigators.	Page 14
Data items	11	List and define all variables for which data were sought and any assumptions and simplifications made.	Page 14
Critical appraisal of individual	12	If done, provide a rationale for conducting a critical appraisal of included sources of	N/A

SECTION	ITEM	PRISMA-ScR CHECKLIST ITEM	REPORTED ON PAGE #
sources of evidence§		evidence; describe the methods used and how this information was used in any data synthesis (if appropriate).	
Synthesis of results	13	Describe the methods of handling and summarizing the data that were charted.	Page 14
**RESULTS**			
Selection of sources of evidence	14	Give numbers of sources of evidence screened, assessed for eligibility, and included in the review, with reasons for exclusions at each stage, ideally using a flow diagram.	Pages 3-4
Characteristics of sources of evidence	15	For each source of evidence, present characteristics for which data were charted and provide the citations.	Page 4, Supplementary material Table S1 & S2
Critical appraisal within sources of evidence	16	If done, present data on critical appraisal of included sources of evidence (see item 12).	N/A
Results of individual sources of evidence	17	For each included source of evidence, present the relevant data that were charted that relate to the review questions and objectives.	Pages 4 -10
Synthesis of results	18	Summarize and/or present the charting results as they relate to the review questions and objectives.	Pages 4-10
**DISCUSSION**			
Summary of evidence	19	Summarize the main results (including an overview of concepts, themes, and types of evidence available), link to the review questions and objectives, and consider the relevance to key groups.	Pages 10- 14
Limitations	20	Discuss the limitations of the scoping review process.	Page 13
Conclusions	21	Provide a general interpretation of the results with respect to the review questions and objectives, as well as potential implications and/or next steps.	Page 14-15
**FUNDING**			
Funding	22	Describe sources of funding for the included sources of evidence, as well as sources of funding for the scoping review. Describe the role of the funders of the scoping review.	Page 20
